# A hidden Markov movement model for rapidly identifying behavioral states from animal tracks

**DOI:** 10.1002/ece3.2795

**Published:** 2017-02-28

**Authors:** Kim Whoriskey, Marie Auger‐Méthé, Christoffer M. Albertsen, Frederick G. Whoriskey, Thomas R. Binder, Charles C. Krueger, Joanna Mills Flemming

**Affiliations:** ^1^ Department of Mathematics and StatisticsDalhousie UniversityHalifaxNSCanada; ^2^ National Institute of Aquatic ResourcesTechnical University of DenmarkCharlottenlundDenmark; ^3^ Ocean Tracking NetworkDalhousie UniversityHalifaxNSCanada; ^4^ Hammond Bay Biological StationDepartment of Fisheries and WildlifeMichigan State UniversityMillersburgMIUSA; ^5^ Center for Systems Integration and SustainabilityMichigan State UniversityEast LansingMIUSA

**Keywords:** behavioral states, Great Lakes Acoustic Telemetry Observation System, movement ecology, Ocean Tracking Network, TMB, swim

## Abstract

Electronic telemetry is frequently used to document animal movement through time. Methods that can identify underlying behaviors driving specific movement patterns can help us understand how and why animals use available space, thereby aiding conservation and management efforts. For aquatic animal tracking data with significant measurement error, a Bayesian state‐space model called the first‐Difference Correlated Random Walk with Switching (DCRWS) has often been used for this purpose. However, for aquatic animals, highly accurate tracking data are now becoming more common. We developed a new hidden Markov model (HMM) for identifying behavioral states from animal tracks with negligible error, called the hidden Markov movement model (HMMM). We implemented as the basis for the HMMM the process equation of the DCRWS, but we used the method of maximum likelihood and the R package TMB for rapid model fitting. The HMMM was compared to a modified version of the DCRWS for highly accurate tracks, the DCRWS${}_{\mathrm{NOME}}$, and to a common HMM for animal tracks fitted with the R package moveHMM. We show that the HMMM is both accurate and suitable for multiple species by fitting it to real tracks from a grey seal, lake trout, and blue shark, as well as to simulated data. The HMMM is a fast and reliable tool for making meaningful inference from animal movement data that is ideally suited for ecologists who want to use the popular DCRWS implementation and have highly accurate tracking data. It additionally provides a groundwork for development of more complex modeling of animal movement with TMB. To facilitate its uptake, we make it available through the R package swim.

## 1 Introduction

Animals move to maximize their growth and to enhance their probability of survival and reproduction. Movement is therefore a critical animal behavior that reflects an animal's response to its current biological and physical needs and to its environment. Identifying these underlying drivers of animal movement (behavioral states) is required for understanding how and why animals use available space, and this knowledge informs the management and conservation of both species and ecosystems. In aquatic environments, where direct observation of animal movement and behavioral states is often impossible, researchers are rapidly expanding the use of electronic telemetry for documenting animal movement through time (Hussey et al., 2015).

Satellite telemetry and acoustic positioning systems are common types of telemetry technology for estimating an aquatic animal's location in continuous space and yield time series of locations along an animal's path, usually referred to as tracks. Inferring behavioral states from animal tracks is possible by assuming that different types of movement, and therefore behavioral states, can be reflected by changes in characteristics of an animal's path. For example, while foraging can often be characterized by a tortuous track, a more directed path may suggest traveling between habitats (e.g., Jonsen, Mills Flemming, & Myers, 2005).

Hidden Markov models (HMMs) are a popular tool used to identify behavioral states from animal telemetry data with negligible error (e.g., Morales, Haydon, Frair, Holsinger, & Fryxell, 2004; Langrock, King, Matthiopoulos, Thomas, Fortin, & Morales, 2012). HMMs are a large class of models distinguished in the most general case by a set of observations that depend on an unobserved, underlying Markov process (Zucchini, MacDonald, & Langrock, 2016). In the context of animal movement, the latent Markov process is used to model the discrete behavioral states of interest, while the set of observations follow a movement process that can also be Markovian. The observations can consist of either location data (e.g., Jonsen et al., 2005) or metrics derived from the observed track, such as turning angles and step lengths (e.g., Morales et al., 2004; Langrock et al., 2012). While current HMMs can be fitted rapidly using maximum‐likelihood (ML) methods, with the exception of the formulation of Pedersen, Patterson, Thygesen and Madsen (2011), they are unable to account for measurement error associated with the technology used to obtain animal tracks. For those tracks measured with error, state‐space models (SSMs) provide a more accurate and reliable method for identifying behavioral states, but are typically fitted using comparatively slow Bayesian methods such as Markov chain Monte Carlo (MCMC) sampling because of large numbers of random effects (e.g., Jonsen et al., 2005; McClintock, King, Thomas, Matthiopoulos, McConnell, & Morales, 2012; Jonsen, 2016). Therefore, the ideal tool for identifying behavioral states from animal tracks should incorporate features of both HMM and SSM implementations, such that measurement error can be accounted for within a ML framework that keeps the computational burden of estimation relatively small.

One particular SSM that has proven its utility through a wide range of applications on different species is the Bayesian first‐Difference Correlated Random Walk with Switching (DCRWS) SSM of Jonsen et al. (2005). This model has been previously used to quantify foraging behavior in cetaceans (Bailey, Mate, Palacios, Irvine, Bograd, & Costa, 2009; Irvine et al., 2014), pinnipeds (Breed, Jonsen, Myers, Bowen, & Leonard, 2009; Harwood, Smith, Auld, Melling, & Yurkowski, 2015), turtles (González Carman et al. 2012; Hart, Lamont, Fujisaki, Tucker, & Carthy, 2012), sea birds (Reid, Ronconi, Cuthbert, & Ryan, 2014), and manta rays (Graham et al., 2012). Furthermore, it has been used to determine migration corridors (Prieto, Silva, Waring, & Gonçalves, 2014), to estimate intraspecific competition (Breed, Bowen, & Leonard, 2013), predator–prey relationships (Fitzpatrick, Thums, Bell, Meekan, Stevens, & Barnett, 2012), and site fidelity (Block et al., 2011), and to inform management and conservation of protected regions (Block et al., 2011; Maxwell et al., 2011; Graham et al., 2012).

Here, we introduce a new HMM for estimating behavioral states from highly accurate animal tracks which is similar to the DCRWS, but does not account for measurement error. We directly implement the process equation of the DCRWS as the basis for our HMM, but we adjust the model for fitting within a ML framework to allow for rapid estimation. Model fitting and parameter estimation are performed using the R package TMB (Kristensen, Nielsen, Berg, & Skaug, 2016), which has previously shown great promise for analyzing animal tracking data (Albertsen, Whoriskey, Yurkowski, Nielsen, & Mills Flemming, 2015; Auger‐Méthé et al. 2017). We make this model, which we entitle the hidden Markov movement model (HMMM), available through the R package swim (see supplementary material). To demonstrate the accuracy and applicability of the HMMM, we apply it to simulated animal tracks and to real tracks from multiple aquatic species. We additionally compare our HMMM results to those obtained using its Bayesian counterpart and to results from the moveHMM package (Michelot, Langrock, & Patterson, 2016). We assess the advantages and disadvantages of each approach by comparing their computational efficiency, accuracy, and sequences of behavioral states.

## 2 Methods

### 2.1 The DCRWS movement process

The DCRWS is a SSM that estimates the true locations, behavioral states, and parameters of a movement process from an Argos satellite system track (Jonsen et al., 2005). Given the true location $x_{t}$ at time *t*, the process equation of the DCRWS is a correlated random walk on the first differences of the true locations, $d_{t}=x_{t}-x_{t-1}$:

(1) $$d_{t}=\gamma_{b_{t-1}}T\left(\theta_{b_{t-1}}\right)d_{t-1}+N_{2}\left(0,\Sigma \right)$$

$$T\left(\theta_{b_{t-1}}\right)=\left(\begin{array}{cc} \cos(\theta_{b_{t-1}}) & -\sin(\theta_{b_{t-1}})\\ \sin(\theta_{b_{t-1}}) & \cos(\theta_{b_{t-1}}) \end{array}\right)\;\Sigma =\left(\begin{array}{cc} \sigma_{\mathrm{lon}}^{2} & \rho \sigma_{\mathrm{lon}}\sigma_{\mathrm{lat}}\\ \rho \sigma_{\mathrm{lon}}\sigma_{\mathrm{lat}} & \sigma_{\mathrm{lat}}^{2} \end{array}\right)$$

The stochastic term in the movement process is a bivariate Gaussian (N${}_{2}$) with mean 0 and covariance matrix Σ, where $\sigma_{\mathrm{lat}}$ and $\sigma_{\mathrm{lon}}$ are the standard deviations in the latitude and longitude axes, respectively, and ρ is the correlation between the two axes. Like Breed et al. (2012), we assume here that ρ = 0, implying that the stochasticity in the longitudinal and latitudinal axes are independent of each other. The parameter $\gamma_{b_{t-1}}$ describes the autocorrelation in both direction and speed, and $T(\theta_{b_{t-1}})$ is the rotational matrix through space given the turning angle $\theta_{b_{t-1}}$. Multiple values are possible for $\gamma_{b_{t-1}}$ and $\theta_{b_{t-1}}$, and these parameter values are dependent on the behavioral state at time *t*−1, that is, $b_{t-1}$. This dependence provides the mechanism for distinguishing between multiple behavioral states at each location. Typically of interest are two states: the first is directed movement characterized by traveling in the same direction (θ ≈ 0) and at a similar, high speed (γ > 0.5), and the second is tortuous movement characterized by frequent course reversals (θ ≈ π) at dissimilar, slower speeds (γ < 0.5). Parameter sets for each state are identified with the appropriate subscript, either 1 or 2.

### 2.2 The HMMM

Our HMMM uses the movement process described by (1), but instead of using a Bayesian framework like Jonsen et al. (2005), we employ a ML framework and fit the process equation as a HMM via TMB, which requires that the likelihood function be coded in the C++ programming language. The probability distribution of the movement process is conditional on the assumed behavioral states $b_{t}$ and is given by

(2) $$f\left(d_{t}|b_{t-1}\right)∼N_{2}\left(\gamma_{b_{t-1}}T\left(\theta_{b_{t-1}}\right)d_{t-1},\Sigma \right).$$

The likelihood of the HMMM is that of a HMM (Zucchini et al., 2016):

(3) $$\delta^{\prime }P\left(d_{1}\right)AP\left(d_{2}\right)A\cdots P\left(d_{t-1}\right)AP\left(d_{t}\right)1.$$

Assuming two behavioral states, the 2×1 vector δ contains the initial probabilities of being in each state. **A** is a 2×2 transition probability matrix containing the switching probabilities $\alpha_{i,j}$ that describe the probability of switching from state *i* at time *t*−1 to state *j* at time *t*. Because the rows of **A** sum to 1, we need only estimate two switching probabilities instead of four; we choose to estimate $\alpha_{1,1}$ and $\alpha_{2,1}$. **P** is a 2×2 diagonal matrix with diagonal entries equal to ($f(d_{t}|b_{t-1}=1)$, $f(d_{t}|b_{t-1}=2)$), that is, the probabilities of being at the observed locations given each behavioral state as described by the movement process. **1** is a 2×1 vector of ones. We estimate the parameters of the movement process directly from the likelihood within TMB and then use the Viterbi algorithm to estimate the unobserved behavioral states (Zucchini et al., 2016).

### 2.3 Data analysis and simulation study

To evaluate the performance of the HMMM, we compared it with two other approaches for estimating behavioral states from animal tracks with negligible measurement error. The first was the switching movement process described by (1), fitted using a Bayesian framework and Markov chain Monte Carlo (MCMC) sampling via rjags (Plummer, 2015). This first model is the DCRWS of Jonsen et al. (2005) modified such that it does not include a measurement equation, and therefore differs from the HMMM solely in implementation (i.e., Bayesian vs. ML inference). Hereafter, we refer to it as the DCRWS${}_{\mathrm{NOME}}$. Although the DCRWS${}_{\mathrm{NOME}}$ has not been fitted before, implementations of the DCRWS for tracking data with minimal errors do exist (Jonsen, 2016), and the DCRWS${}_{\mathrm{NOME}}$ is the most direct implementation of the DCRWS when no measurement error is assumed. To fit the DCRWS${}_{\mathrm{NOME}}$, we used a burn‐in period of 40,000 samples and then sampled 20,000 from the posterior distribution but only kept every 20th sample (thinning). We fitted and compared two MCMC chains to each track to check for convergence. All prior distributions were specified as in the R package bsam (Jonsen et al., 2005) that fits the original DCRWS model, with the exception of those for the error covariance matrix Σ. Instead, by setting ρ = 0, which we believe is more appropriate, we were able to specify separate vague uniform priors on $\sigma_{\mathrm{lon}}$ and $\sigma_{\mathrm{lat}}$ as opposed to using the original Wishart prior on the entire matrix (Jonsen et al., 2005). Parameters and behavioral states were estimated as the posterior medians of the samples from the two chains combined. We additionally fitted a HMM to the turning angles (rad) and step lengths (km) of the animal tracks with the R package moveHMM (Michelot et al., 2016), using a von Mises (mean *μ* and concentration parameter *c*) and Weibull (shape λ and scale parameter *k*) distribution, respectively. Behavioral states were again identified via the Viterbi algorithm, using functions from moveHMM.

We fitted these three models to three animal tracks: (1) a GPS track collected by a Sea Mammal Research Unit head‐mounted Satellite Relay Data Logger (accurate GPS positions acquired when the head surfaces) deployed on an adult male grey seal (*Halichoerus grypus*) at Kouchibouguac National Park, New Brunswick, in 2013; (2) an acoustic Vemco Positioning System (VPS; positions from triangulation of detections from multiple receivers in known locations; Smith, 2013) track of an adult male lake trout (*Salvelinus namaycush*) in northern Lake Huron in 2014; and (3) a light‐based geolocation track recorded by an immature female blue shark (*Prionace glauca*) tagged near Halifax, Nova Scotia, with a Wildlife Computers miniPAT tag in 2014. Because geolocation data can be error‐prone, the track was processed with the Wildlife Computers (2015) GPE3 software (a SSM) to improve positioning accuracy by estimating the true blue shark locations, as suggested by the manufacturers. Although a SSM for geolocation data would have been the ideal approach for analyzing the blue shark data, our approach of fitting HMMs to true location SSM estimates has been previously adopted (e.g., Eckert et al., 2008). Because the data were collected in continuous‐time but all three models assume underlying discrete‐time Markov processes, we had to approximate the locations in discrete‐time and then assume that these were known. We linearly interpolated the data sets over time using a 6‐hr, 15‐min, and 12‐hr time step for the grey seal, lake trout, and blue shark data, respectively, yielding data sets with 1,227, 2,187, and 393 locations. Different time steps were required based on the different temporal resolutions of the tracks.

Additionally, using the parameter estimates from the grey seal HMMM and DCRWS${}_{\mathrm{NOME}}$ fits (Table 1), we conducted a simulation study to formally compare the accuracy of the HMMM and DCRWS${}_{\mathrm{NOME}}$ and compare their results with those obtained using moveHMM. We simulated 50 tracks corresponding to the HMMM from a known parameter set with turning angles $\theta_{1}\;=\;0$ and $\theta_{2}\;=\;\pi$; autocorrelation $\gamma_{1}\;=\;0.8$ and $\gamma_{2}\;=\;0.05$; process error standard deviations $\sigma_{\mathrm{lon}}\;=\;0.07$ and $\sigma_{\mathrm{lat}}\;=\;0.05$; and switching probabilities $\alpha_{1,1}\;=\;0.89$ and $\alpha_{2,1}\;=\;0.20$. These two behavioral states are classically interpreted as transiting ($\theta_{1}$, $\gamma_{1}$) and foraging ($\theta_{2}$, $\gamma_{2}$). We then fitted the HMMM, moveHMM, and the DCRWS${}_{\mathrm{NOME}}$ to each simulated track and calculated the parameter estimates and interval measures of uncertainty for these estimates. For the HMMM and moveHMM, this consisted of the 95% confidence interval based on the standard error estimates. For the DCRWS${}_{\mathrm{NOME}}$, we determined the 95% credible interval as the 2.5% and 97.5% quantiles of the posterior samples. We found the behavioral state error rate, that is, the proportion of states that were incorrectly identified, for each model, and additionally calculated the root mean squared error (RMSE) for each parameter estimate $\hat{\Theta }$ from the HMMM and DCRWS${}_{\mathrm{NOME}}$ fits as

(4) $${\text{RMSE}}_{\Theta }={\left(\frac{1}{n}\sum_{j=1}^{n}{\left(\hat{\Theta }-\Theta \right)}^{2}\right)}^{1/2}$$

**Table 1:** Parameter estimates from three models fitted to a grey seal track. The Lower and Upper columns are the lower and upper bounds of 95% uncertainty intervals around the estimates. These correspond to 95% confidence intervals for the HMMM and moveHMM, and 95% credible intervals for the DCRWS${}_{\mathrm{NOME}}$. The only two parameters in common among all three models are the switching probabilities, $\alpha_{1,1}$ and $\alpha_{2,1}$

Parameter	HMMM			DCRWS${}_{\mathrm{NOME}}$			Parameter	moveHMM		
	Estimate	Lower	Upper	Estimate	Lower	Upper		Estimate	Lower	Upper
$\theta_{1}$	0.022	−0.023	0.066	−0.017	−0.060	0.027	$\mu_{1}$	−0.010	−0.058	0.038
$\theta_{2}$	4.662	2.441	5.835	1.831	0.275	5.980	$\mu_{2}$	0.495	−0.358	1.348
$\gamma_{1}$	0.805	0.753	0.848	0.805	0.759	0.849	$c_{1}$	0.685	0.640	0.730
$\gamma_{2}$	0.055	0.013	0.201	0.048	0.003	0.128	$c_{2}$	0.069	0.002	0.135
$\sigma_{\mathrm{lon}}$	0.071	0.068	0.074	0.071	0.068	0.074	$\lambda_{1}$	2.185	1.977	2.393
$\sigma_{\mathrm{lat}}$	0.050	0.048	0.053	0.050	0.048	0.053	$\lambda_{2}$	0.816	0.757	0.875
							$k_{1}$	15.342	14.381	16.304
							$k_{2}$	3.487	2.878	4.097
$\alpha_{1,1}$	0.890	0.827	0.932	0.885	0.835	0.929		0.876	0.842	0.910
$\alpha_{2,1}$	0.198	0.133	0.285	0.204	0.141	0.292		0.111	0.090	0.158

We were unable to calculate the RMSE for the moveHMM fits because the data were simulated according to the HMMM movement process and the moveHMM implementation does not involve the same parameters.

## 3 Results

### 3.1 Identifying behavioral states

**Figure 1:**
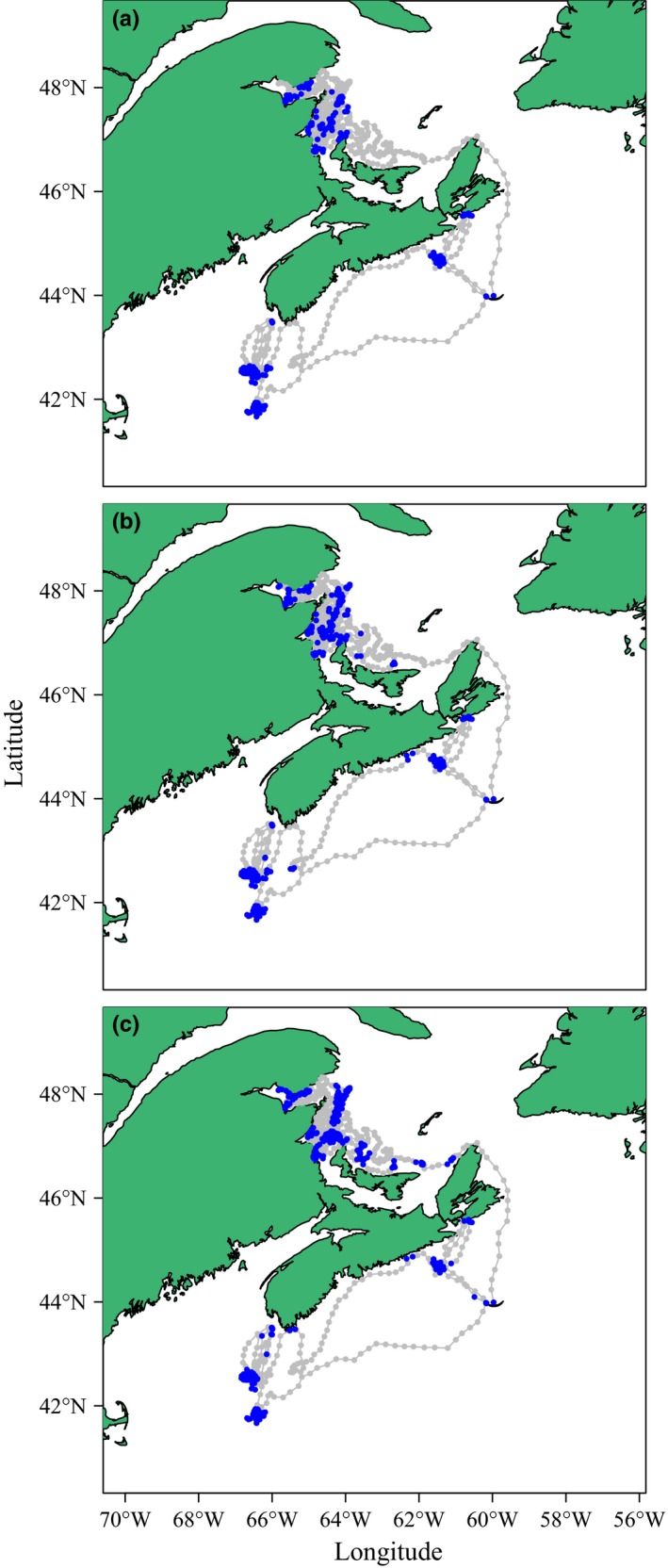
Behavioral states as obtained by fitting the HMMM (panel a), DCRWS_NOME_ (panel b), and moveHMM (panel c) models to the grey seal track. Different behavioral states are indicated by grey (state 1) and blue (state 2)

**Figure 2:**
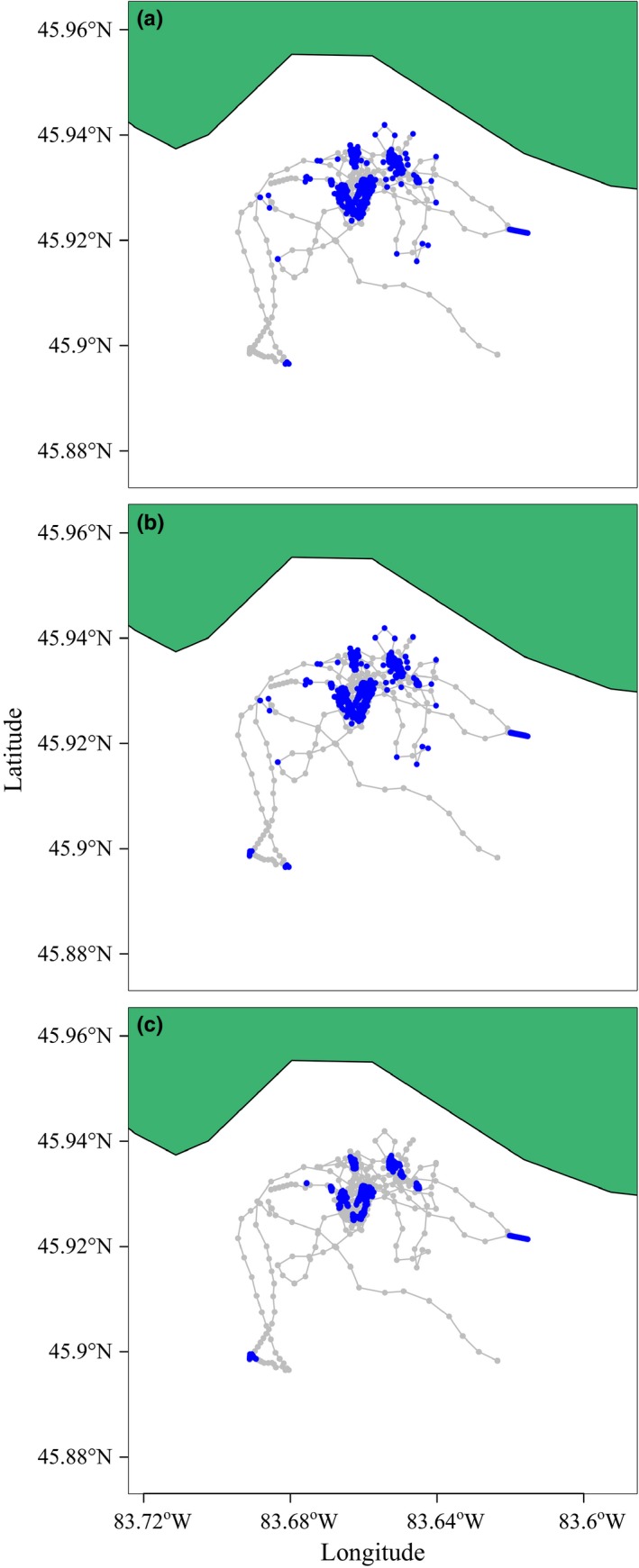
Behavioral states as obtained by fitting the HMMM (panel a), DCRWS_NOME_ (panel b), and moveHMM (panel c) models to the lake trout track. Different behavioral states are indicated by grey (state 1) and blue (state 2)

We applied the HMMM, the DCRWS${}_{\mathrm{NOME}}$, and moveHMM to a grey seal, lake trout, and blue shark track estimated with negligible measurement error. All three models performed similarly and identified two clearly distinct behavioral states for the grey seal and lake trout tracks (Figures 1 and 2). For both animals, HMMM and DCRWS${}_{\mathrm{NOME}}$ parameter estimates were similar except for the tortuous turning angle $\theta_{2}$, which was estimated at a similar distance from the number π but in the opposite direction (i.e., while one was estimated turning slightly to the left, the other was estimated turning to the right; Tables 1 and 2). This, along with the relatively large confidence intervals for $\theta_{2}$, is not unusual because together with small $\gamma_{2}$, it suggests that the animal is exhibiting tortuous movement, in which case the mean turning angle does not have as much influence because the animal is more equally likely to travel in any direction. Switching probabilities ($\alpha_{1,1}$ and $\alpha_{2,1}$) were similar among all three models for the seal track. moveHMM estimated switching probabilities for the lake trout track different from the HMMM and DCRWS${}_{\mathrm{NOME}}$, although the estimated parameters of the three models led to similar decoded behavioral state sequences. For the seal track, the DCRWS${}_{\mathrm{NOME}}$ took 6.4 hr to fit, moveHMM took 0.9 s, and the HMMM took 0.06 s. For the lake trout data, the DCRWS${}_{\mathrm{NOME}}$ took 9.4 hr to fit, moveHMM took 1.8 s, and the HMMM took 0.16 s.

**Table 2:** Parameter estimates from three models fitted to a lake trout track. The Lower and Upper columns are the lower and upper bounds of 95% uncertainty intervals around the estimates. These correspond to 95% confidence intervals for the HMMM and moveHMM, and 95% credible intervals for the DCRWS${}_{\mathrm{NOME}}$. The only two parameters in common among all three models are the switching probabilities, $\alpha_{1,1}$ and $\alpha_{2,1}$

Parameter	HMMM			DCRWS${}_{\mathrm{NOME}}$			Parameter	moveHMM		
	Estimate	Lower	Upper	Estimate	Lower	Upper		Estimate	Lower	Upper
$\theta_{1}$	−0.118	−0.155	−0.082	0.119	0.084	0.155	$\mu_{1}$	0.021	−0.041	0.083
$\theta_{2}$	2.687	2.277	3.113	3.603	3.206	4.088	$\mu_{2}$	−0.746	−2.042	0.447
$\gamma_{1}$	0.821	0.786	0.851	0.821	0.788	0.853	$c_{1}$	3.123	2.488	3.763
$\gamma_{2}$	0.128	0.083	0.191	0.123	0.075	0.177	$c_{2}$	0.113	0.033	0.238
$\sigma_{lon}$	0.001	0.001	0.001	0.001	0.001	0.001	$\lambda_{1}$	2.324	2.119	2.550
$\sigma_{lat}$	0.001	0.001	0.001	0.001	0.001	0.001	$\lambda_{2}$	0.838	0.786	0.894
							$k_{1}$	16.128	15.274	17.029
							$k_{2}$	4.084	3.621	4.606
$\alpha_{1,1}$	0.645	0.578	0.707	0.643	0.576	0.705		0.853	0.811	0.887
$\alpha_{2,1}$	0.288	0.212	0.377	0.289	0.214	0.384		0.102	0.077	0.132

**Figure 3:**
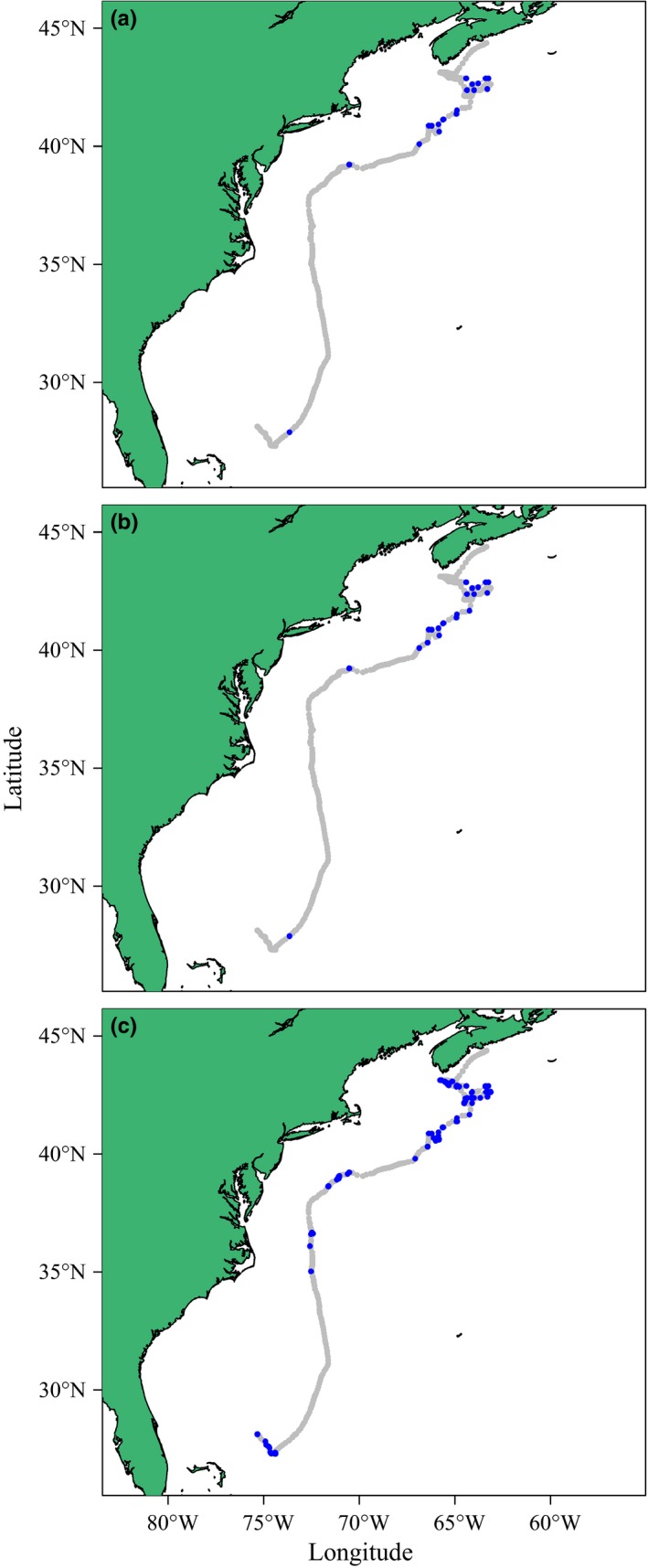
Behavioral states as obtained by fitting the HMMM (panel a), DCRWS_NOME_ (panel b), and moveHMM (panel c) models to the blue shark track. Different behavioral states are indicated by grey (state 1) and blue (state 2)

All three models identified two states from the blue shark track, although half of the switching probabilities estimated by moveHMM differed greatly from those estimated by the HMMM and DCRWS${}_{\mathrm{NOME}}$ (Table 3), and this led to different state sequences (Figure 3). Specifically, all three models estimated a high probability of remaining in state 1, $\alpha_{1,1}$, but moveHMM estimated a low probability of switching from state 2 to state 1, $\alpha_{2,1}$, while the DCRWS${}_{\mathrm{NOME}}$ and HMMM estimated a high $\alpha_{2,1}$. The switching probabilities of the HMMM and DCRWS${}_{\mathrm{NOME}}$ therefore led to state sequences containing long stretches of state 1 interspersed with short (length 1 or 2) stretches of state 2. By contrast, moveHMM estimated a state sequence with longer stretches of both behavioral states. While the DCRWS${}_{\mathrm{NOME}}$ took 1.7 hr to fit to the blue shark track, moveHMM took 1.2 s, and the HMMM took 0.02 s.

**Table 3:** Parameter estimates from three models fitted to a blue shark track. The Lower and Upper columns are the lower and upper bounds of 95% uncertainty intervals around the estimates. These correspond to 95% confidence intervals for the HMMM and moveHMM, and 95% credible intervals for the DCRWS${}_{\mathrm{NOME}}$. The only two parameters in common among all three models are the switching probabilities, $\alpha_{1,1}$ and $\alpha_{2,1}$. Because this track had some step lengths equal to zero, the two parameters $\zeta_{1}$ and $\zeta_{2}$ were used to estimate zero‐inflation for each behavior when using moveHMM

Parameter	HMMM			DCRWS${}_{\mathrm{NOME}}$			Parameter	moveHMM		
	Estimate	Lower	Upper	Estimate	Lower	Upper		Estimate	Lower	Upper
$\theta_{1}$	−0.021	−0.070	0.027	0.013	−0.040	0.062	$\mu_{1}$	−0.003	−0.025	0.019
$\theta_{2}$	0.528	0.232	1.131	−0.881	−0.006	0.157	$\mu_{2}$	0.013	−0.273	0.300
$\gamma_{1}$	0.923	0.846	0.963	0.932	0.873	0.987	$c_{1}$	40.323	30.556	50.107
$\gamma_{2}$	0.289	0.199	0.400	0.303	0.188	0.423	$c_{2}$	0.949	0.616	1.302
$\sigma_{\mathrm{lon}}$	0.045	0.042	0.049	0.046	0.043	0.049	$\lambda_{1}$	1.806	1.608	2.029
$\sigma_{\mathrm{lat}}$	0.042	0.039	0.045	0.042	0.038	0.045	$\lambda_{2}$	1.069	0.927	1.232
							$k_{1}$	11.816	10.872	12.842
							$k_{2}$	6.610	5.255	8.314
							$\zeta_{1}$	0.029	0.014	0.059
							$\zeta_{2}$	0.035	0.013	0.091
$\alpha_{1,1}$	0.904	0.794	0.958	0.880	0.732	0.955		0.841	0.778	0.888
$\alpha_{2,1}$	0.722	0.385	0.915	0.742	0.437	0.925		0.320	0.211	0.454

### 3.2 Simulation Study

**Figure 4:**
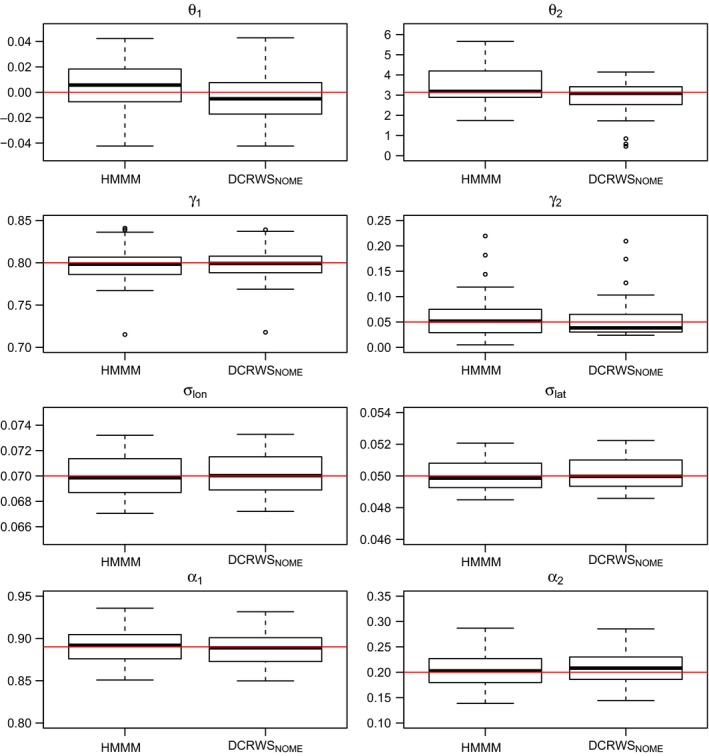
Boxplots of parameter estimates obtained from fitting the HMMM and the DCRWS_NOME_ to 50 simulated tracks

We simulated 50 tracks from the HMMM with a set of parameters representative of the grey seal track. The HMMM and DCRWS${}_{\mathrm{NOME}}$ provided accurate estimates of the model parameters (Figure 4), but the DCRWS${}_{\mathrm{NOME}}$ had a smaller average (over the parameters) RMSE (0.120 vs. 0.140; Table 4). The RMSEs for individual parameters were similar (within 0.01) between the two models with the exception of $\theta_{2}$, where the RMSE of the DCRWS${}_{\mathrm{NOME}}$ was smaller by 0.149 (Table 4). The DCRWS${}_{\mathrm{NOME}}$ additionally had the smallest behavioral state error rate (0.175) which differed from the HMMM and moveHMM by approximately 1.5% (0.189) and 18.7% (0.362), respectively. Finally, the average time needed to fit the DCRWS${}_{\mathrm{NOME}}$ was 5.10 hr, while moveHMM took 1.2 s and the HMMM took 0.08 s.

**Table 4:** Parameter results from the simulation study (*n* = 50) comparing the HMMM to the DCRWS${}_{\mathrm{NOME}}$. The Lower and Upper columns correspond to the 95% confidence and credible intervals for the HMMM and the DCRWS${}_{\mathrm{NOME}}$, respectively. The Estimate, Lower, and Upper columns are averages taken over all simulations. The RMSE columns contain the root mean squared errors

Parameter	True value	HMMM				DCRWS${}_{\mathrm{NOME}}$			
		Estimate	Lower	Upper	RMSE	Estimate	Lower	Upper	RMSE
$\theta_{1}$	0	0.004	−0.035	0.042	0.020	−0.004	−0.043	0.035	0.020
$\theta_{2}$	π	3.433	1.599	4.730	0.978	2.931	0.483	5.400	0.829
$\gamma_{1}$	0.80	0.797	0.752	0.836	0.021	0.797	0.756	0.839	0.021
$\gamma_{2}$	0.05	0.060	0.018	0.321	0.044	0.052	0.007	0.138	0.037
$\sigma_{lon}$	0.07	0.070	0.067	0.073	0.002	0.070	0.067	0.073	0.002
$\sigma_{lat}$	0.05	0.050	0.048	0.052	0.001	0.050	0.048	0.052	0.001
$\alpha_{1,1}$	0.89	0.890	0.844	0.924	0.018	0.886	0.842	0.922	0.018
$\alpha_{2,1}$	0.20	0.205	0.140	0.290	0.034	0.208	0.143	0.293	0.033

## 4 Discussion

We have shown that the HMMM is a fast and reliable tool for estimating behavioral states from animal tracking data that contain negligible error. Our simulation study demonstrated the accuracy of the HMMM for estimating both the states and model parameters. Our use of data from different species and derived by different telemetry systems demonstrated the wide‐ranging applicability of the HMMM. Coupled with the existing documentation of the DCRWS (more than 45 papers using this model), we suspect that the HMMM will be an easily interpretable tool for ecologists who are interested in implementing the DCRWS and have highly accurate data, which has the advantage over current methods with the DCRWS of being fast to fit (on the order of seconds) and avoiding convergence issues with MCMC samplers. HMMM implementation is available through the R package swim.

The HMMM, DCRWS${}_{\mathrm{NOME}}$, and moveHMM all identified two behavioral states from the grey seal track, consistent with previous analyses (Jonsen et al., 2005; Breed et al., 2009; Breed, Bowen, & Leonard, 2011). Grey seal tracks from Atlantic Canada typically show clear bouts of directed and tortuous movement and have been previously analyzed with the DCRWS (e.g., Jonsen et al., 2005); therefore, an Atlantic Canada grey seal GPS track provided an ideal test for our study. For grey seals, tortuous movement is classically interpreted as foraging, while directed movement is often regarded as traveling between foraging patches. The models identified several bouts of foraging behavior in the Northwest Atlantic and in the Gulf of Saint Lawrence, specifically off the coasts of Nova Scotia, Prince Edward Island, New Brunswick, and Gaspésie, areas of high biological productivity that are consistent with those previously identified as grey seal foraging areas (Breed et al., 2009).

With the HMMM, DCRWS${}_{\mathrm{NOME}}$, and moveHMM, we identified two behavioral states within the lake trout track. The Drummond Island lake trout population spawn primarily at nighttime on rock rubble reefs in association with submerged drumlins (Riley et al., 2014; Binder et al., 2015). Lake trout show multiple behaviors characterized by tortuous movement, including spawning on the reefs. For example, lake trout (particularly males) often aggregate on the spawning reefs in the weeks leading up to spawning, a behavior known as staging (Muir, Blackie, Marsden, & Krueger, 2012). Because egg surveys have verified that no spawning occurs in some locations where our models identified tortuous behavior (T. Binder, unpublished observations), we believe the models are distinguishing, more generally, reef and non‐reef behaviors. Being able to mathematically distinguish between reef and non‐reef behaviors can allow for identification of key lake trout habitats for conservation such as spawning sites in places where direct observation is difficult. Furthermore, by building a dependence of the HMMM on one or more covariates, it may be possible to more acutely identify spawning behavior. For example, because the Drummond Island lake trout tend to spawn at night close to the substrate, time of day and lake trout depth (which is often recorded by positioning systems such as the VPS) may provide sufficient additional information for the HMMM to distinguish spawning behavior from other reef‐associated behaviors. One possible way to achieve this is by allowing the switching probabilities of the HMMM to depend on these covariates in a linear fashion (as in, e.g., Bestley, Jonsen, Hindell, Guinet, & Charrassin, 2012; Michelot et al., 2016). Additionally, an extension to the HMMM which could estimate more than two behavioral states may be able to distinguish reef from spawning behavior. We chose to model only two states so that we could more directly compare results of the HMMM to our implementation of the original DCRWS (the DCRWS${}_{\mathrm{NOME}}$); however, the HMMM should be directly extendible.

When fitted to the blue shark track, moveHMM produced different state sequences than the HMMM and DCRWS${}_{\mathrm{NOME}}$, as moveHMM estimated longer stretches of behavioral state 2 than either of the other models. This is likely because moveHMM models the distributions of the turning angles and step lengths calculated from an animal path, which is fundamentally different from the movement process of the HMMM and DCRWS${}_{\mathrm{NOME}}$. Furthermore, McClintock et al. (2014) showed that the continuous‐time analog to the movement process introduced by Jonsen et al. (2005) and modeled by the HMMM has step lengths and bearings that are correlated, whereas the step lengths and bearings of the process modeled by McClintock et al. (2012) (close to that of moveHMM) are uncorrelated. moveHMM identified two behaviors that were distinguished primarily by different step lengths, and therefore traveling speeds, with state 1 characterized by longer step lengths and faster speeds, and state 2 characterized by slower movement. The HMMM and DCRWS${}_{\mathrm{NOME}}$ identified two behaviors that were distinguished by high (state 1) and low (state 2) autocorrelations, or how related the speed at time *t* was to the speed at time *t*−1. By modeling autocorrelation, the HMMM and DCRWS${}_{\mathrm{NOME}}$ were able to directly estimate persistence in animal movement, which reflected an animal's choice to move. However, it is possible that the shorter sequences of state 2 were identified by the HMMM and DCRWS${}_{\mathrm{NOME}}$ because the behaviors they were trying to estimate occurred on a finer timescale than was modeled, which could make biological interpretation of these states difficult.

Our simulation study results suggested that while the DCRWS${}_{\mathrm{NOME}}$ was slightly more accurate than the HMMM, the difference was marginal. The two models performed similarly while estimating model parameters with the exception of $\theta_{2}$, which the DCRWS${}_{\mathrm{NOME}}$ more accurately estimated. This result is likely explained by the rather informative priors on $\theta_{1}$ and $\gamma_{1}$ when fitting the DCRWS${}_{\mathrm{NOME}}$. The DCRWS${}_{\mathrm{NOME}}$ also more accurately estimated the behavioral states, an unsurprising result because while the DCRWS${}_{\mathrm{NOME}}$ directly estimates these random effects from the posterior likelihood, the HMMM uses a post hoc global decoding algorithm (the Viterbi algorithm) to identify the most likely sequence of states given the ML parameter estimates. Predictably, moveHMM had the highest behavioral state error rate of the three approaches, likely because it was fitted to simulated data from a movement process not equivalent to its own. Finally, the HMMM was the fastest model to fit, with moveHMM and the DCRWS${}_{\mathrm{NOME}}$ taking on average 15 times and 229,500 times longer to fit than the HMMM, respectively. Quicker fits of the DCRWS${}_{\mathrm{NOME}}$ may be achieved by reducing burn‐in and sampling sizes of the MCMC, but they would still take orders of magnitude longer and may be less accurate. We chose these sizes based on prior experience with fitting the DCRWS, and to try to ensure convergence of the MCMC chains during the simulation study.

Our HMMM is a major advance in using TMB to solve animal movement problems. Highly accurate data are becoming more common in the marine realm, and the HMMM, as implemented through the R package swim, provides a fast and reliable tool for making meaningful inference from animal movement data. Fast methods for analyzing data will become more important as larger data sets are collected. The HMMM therefore additionally provides a baseline method for movement modeling in TMB that can be further developed for more specific and nontrivial animal movement problems such as determining relationships between movement and environmental covariates, or accounting for measurement error.

## Conflict of Interest

None declared.
